# Microstructural Analysis of Recast Layer Thickness and Microcrack Formation During EDM of Hastelloy C-22 with Different Graphite Electrodes

**DOI:** 10.3390/ma18235338

**Published:** 2025-11-27

**Authors:** Rafał Nowicki, Rafał Świercz

**Affiliations:** Institute of Manufacturing Technology, Faculty of Mechanical and Industrial Technology, Warsaw University of Technology, Narbutta 85, 02-524 Warsaw, Poland; rafal.swiercz@pw.edu.pl

**Keywords:** electrical discharge machining (EDM), recast layer thickness, microcracks, Hastelloy C-22, graphite electrodes, grain size

## Abstract

Electrical discharge machining is a non-conventional shaping technique applied to electrically conductive, difficult-to-machine alloys, such as Hastelloy C-22. This study investigates the influence of graphite electrode properties and key machining parameters on the average thickness of the recast layer under positive polarity. Two POCO graphite electrodes with different grain sizes—AF-5 (1 μm) and S-180 (10 μm)—were used to examine the effects of discharge current, pulse duration, and interval on recast layer formation. Metallographic analyses measured layer thickness and observed microstructural defects, including microcracks. Results show that discharge current and pulse duration are the primary factors controlling recast layer thickness, with higher currents and longer pulses producing thicker layers due to resolidification of molten material remaining in the plasma-formed crater. The coarser S-180 electrode caused slightly higher microcrack density and greater thickness variations due to its lower electrical resistivity. Pulse interval mainly influenced discharge stability and debris removal, with minimal effect on average layer thickness. Statistical regression models were developed to quantify the relationships between machining parameters, electrode type, and recast layer thickness, providing predictive tools for selecting optimal conditions. These findings contribute to improving surface integrity and process control in electrical discharge machining of nickel-based alloys.

## 1. Introduction

Electrical Discharge Machining (EDM) is a non-traditional process that shapes conductive materials using controlled electrical discharges instead of mechanical cutting. Each spark forms a high-temperature plasma that melts and vaporises small areas of the workpiece. When the discharge ends, the molten material cools in the dielectric, leaving the typical cratered surface [1,2,3,4,5].

The dielectric fluid in EDM provides insulation, stabilises the discharge, and removes debris, directly affecting the course of machining. Pulse parameters such as current, duration, and voltage determine the thermal load and the resulting surface quality. This makes EDM well-suited for hard-to-machine alloys, such as Hastelloy C-22, enabling the precise shaping of complex geometries. Hastelloy C-22 is widely used in chemical, aerospace, and marine industries due to its exceptional corrosion resistance, thermal stability, and mechanical strength. Investigating EDM of this alloy is crucial because surface integrity has a direct impact on the performance and longevity of components in demanding operational environments [6,7,8,9,10,11,12,13,14,15].

The material removal mechanism in EDM is inherently thermal, and thus, the process not only affects surface topography but also modifies the subsurface microstructure. During each discharge, a portion of the molten material resolidifies on the surface, forming a thin layer known as the recast layer or white layer [16]. This layer typically exhibits altered microstructure, residual stresses, and the presence of microcracks or pores. The properties and thickness of the recast layer depend strongly on the discharge energy and thermal gradients experienced during processing. High discharge currents and prolonged pulse durations typically increase the energy input, leading to thicker recast layers and more pronounced thermal damage. Conversely, lower energy parameters may yield smoother surfaces with thinner modified zones, but at the expense of reduced material removal rates. These surface modifications also influence functional properties such as corrosion resistance, wettability, and wear behaviour, which are critical for the practical applications of EDM-processed components [17,18,19,20,21]. Different EDM discharge conditions can influence surface functionality: higher energy discharges may create thicker recast layers, which can reduce corrosion resistance, whereas optimised discharge parameters can improve surface wettability or reduce friction and wear. Therefore, understanding the relationship between discharge parameters and surface properties is essential for both scientific study and industrial application [22,23,24].

During EDM, high transient temperatures from electrical sparks cause intense melting and vaporization in the discharge zone. Rapid quenching by the dielectric fluid forms distinct layers with different microstructures, typically including the recast layer (white layer), the heat-affected zone (HAZ), and the tempered or unaffected substrate [25,26,27,28,29].

The recast layer forms from molten material that resolidifies on the surface rather than being removed by the dielectric. Its thickness and morphology are governed by discharge energy, pulse duration, polarity, dielectric properties, and cooling rate. Rapid solidification often produces a dense or partially amorphous structure with nano-crystalline regions and oxides. Microstructural studies show columnar or dendritic grains near the surface, with finer equiaxed structures deeper in the layer. In some alloys, such as nickel-based or titanium alloys, oxygen enrichment and elemental redistribution are also observed [26,30,31].

Beneath the resolidified zone lies the heat-affected zone, where the material is not melted but undergoes significant thermal modification. This region typically exhibits grain coarsening or partial recrystallisation due to prolonged exposure to elevated temperatures. The microstructure of the HAZ frequently consists of tempered or reformed martensite, depending on the alloy system [32,33].

Microcracks commonly form within the recast layer due to severe thermal gradients, rapid solidification, and differential contraction with the underlying material. Tensile residual stresses and the brittle nature of resolidified material promote crack initiation and propagation. Crack density and depth increase with higher discharge energy, longer pulses, and in materials with higher hardness or finer grain structures. Microcrack formation can be mitigated by using moderate discharge currents, shorter pulse durations, and appropriate pulse intervals, which reduce thermal gradients while maintaining stable debris removal [25,32,34].

Numerous studies have focused on understanding the formation and characteristics of the recast layer after EDM [35,36,37,38,39,40,41,42,43,44]. It has been demonstrated that the solidification rate, cooling mechanism, and machining parameters have a significant impact on the microstructural evolution within this layer. Soleymani et al. [45] demonstrated that increasing the rotational speed in electrical discharge turning (EDT) of WC-Co composites resulted in a significant reduction in both the recast layer thickness and surface roughness. This effect was attributed to improved flushing efficiency in the machining gap and a lower concentration of thermal energy from electrical discharges. Similar dependencies were observed by Dwivedi [46]. Dhaker et al. [47] developed a data-driven model to predict and minimize the recast layer thickness during electric discharge drilling of Inconel 718. The results showed that the recast layer strongly depends on key process parameters, such as discharge current and time, as well as flushing pressure, allowing for the optimisation of surface integrity in aerospace components. Wang et al. [27] investigated the formation mechanism and morphology of the recast layer in micro electrical discharge machining. The results showed that the recast layer thickness and surface features are strongly influenced by discharge energy, voltage, and capacitance, with lower capacitance and higher voltage helping to reduce the recast layer and improve surface quality. Barman et al. [48] reported that a higher material removal rate worsens the surface finish, as the recast layer is porous, contains debris, and exhibits microcracks. Rajendran et al. [49] demonstrated that the formation of microcracks is primarily related to the properties of the base material, the wear of the tool electrode, and the thickness of the recast layer. Chen et al. [50] proved that the type of tool electrode material used has a significant influence on the formation of the recast layer and the propagation of microcracks.

Based on the above literature, this study extends the author’s previous work [51,52] by addressing key gaps in the understanding of EDM of nickel-based superalloys, particularly Hastelloy C-22. In industrial applications, ensuring high surface integrity is critical because the thickness and uniformity of the recast layer directly affect corrosion resistance and mechanical performance. While the recast layer has been widely studied, little is known about how the microstructure and electrical properties of graphite electrodes affect the thermal conditions in the sparking gap and, consequently, the formation of the recast layer. Comparative studies of industrial POCO graphite electrodes with significantly different grain sizes are especially scarce.

The main objective of this work is to investigate how EDM parameters and electrode properties affect the average recast layer thickness (WLT) and the occurrence of microcracks in Hastelloy C-22. To achieve this aim, the study focuses on four tasks: (1) systematically varying discharge current, pulse duration, and pulse interval to evaluate their effect on WLT; (2) comparing graphite electrodes of different grain sizes and resistivities to assess their influence on surface integrity; (3) analyzing microstructural defects, including microcracks, using metallographic techniques; and (4) developing predictive regression models linking electrode and process parameters to WLT. The novelty of this work lies in connecting electrode material properties to discharge energy and recast layer formation, providing insights for optimising EDM conditions to improve both surface quality and process efficiency.

## 2. Materials and Methods

### 2.1. Study Objective and Scope

The primary objective of this study is to investigate the influence of graphite electrode properties and electrical discharge machining parameters on the average thickness of the recast layer (WLT) in Hastelloy C-22 after EDM with positive polarity. Specifically, the research aims to evaluate how the discharge current (*I*_c_), discharge time (*t*_on_), and time interval (*t*_off_), as well as the grain size and physical characteristics of graphite electrodes: POCO AF-5 and POCO S-180 (POCO Graphite, Inc., Decatur, TX, USA), affect the formation, uniformity, and microstructural integrity of the recast layer. Graphite electrodes were selected due to their low cost, good machinability, and stable performance, which explains their increasingly widespread industrial use compared with copper electrodes. Their distinct electrical and thermal properties, along with differences in grain size and resistivity, make graphite an important material for analysing how electrode characteristics influence recast layer thickness and microcrack formation. Additionally, the study aims to develop predictive statistical models that describe the relationships between process parameters, electrode type, and recast layer thickness, providing a basis for optimising EDM conditions to improve surface quality and minimise defects.

### 2.2. Tool Electrode Material

Two grades of POCO graphite electrodes with significantly different grain sizes were employed: AF-5 with an average grain size of 1 μm and S-180 with a grain size of 10 μm. The electrodes were cut into blocks measuring 12 × 12 × 25 mm using a WEDM machine Robofil 440 SLP (GF Solutions, Bienne, Switzerland). Before machining, both the electrodes and workpiece surfaces were carefully lapped and polished to ensure uniform contact and reproducible machining conditions. Key physical properties of the graphite electrodes are presented in Table 1.

### 2.3. Workpiece Material

The experimental investigation focused on analyzing the effect of electrical discharge machining (EDM) parameters and tool electrode material on the thickness of the recast layer (WLT) formed on Hastelloy C-22. Hastelloy C-22 is a nickel-chromium-molybdenum alloy. The material was selected because of its exceptional corrosion resistance, high hardness, and difficulty in conventional machining. The alloy’s superior resistance to oxidation, pitting, crevice corrosion, and stress corrosion makes it widely used in chemical, aerospace, and marine applications. Cylindrical samples with dimensions of 12 mm × 2.5 mm were prepared for the experiments. Table 2 provides the chemical composition of Hastelloy C-22.

### 2.4. EDM Equipment and Machining Setup

All experiments were conducted on a Charmilles Form 2-LC ZNC die-sinking EDM machine (GF Solutions, Bienne, Switzerland), equipped with a transistor-based pulse generator that independently controls discharge current (*I*_c_), discharge time (*t*_on_), and pulse interval (*t*_off_). A free-kinematics machining strategy was used, where only the frontal surface of the electrode engaged the workpiece. This method eliminated side-wall effects and maintained a consistent spark gap throughout the experiment. Both the workpiece and electrodes were fully immersed in a kerosene-based dielectric fluid (EDM fluid 108 MP-SE) to ensure stable discharge conditions and facilitate debris removal.

Electrical signals, including voltage and current, in the spark gap were monitored in real-time using a voltage probe and a precision current shunt. This setup enabled accurate recording of machining conditions and verification of process stability during the experiments.

### 2.5. Experimental Plan

Before establishing the experimental research plan, preliminary studies were conducted to define stable ranges of EDM parameters. Unstable discharges, often caused by excessively long pulse durations combined with short pulse intervals, led to uneven deposition of resolidified material. These phenomena led to the formation of short-circuit impulses that damaged (burned) the machined surface. Discharge current was found to directly influence the thickness of the recast layer and surface integrity, as higher currents increase the thermal energy delivered to the surface. Similarly, discharge time affects the extent of localised melting, while pulse interval determines the efficiency of debris removal from the spark gap and maintains stable conditions in the machining zone.

The primary experimental research investigated the effects of discharge current (*I*_c_), discharge time (*t*_on_), and time interval (*t*_off_) on the thickness of the recast layer (WLT). Furthermore, the study examined the influence of graphite electrodes, considering their physical properties and grain sizes (AF-5 and S-180), on the machining outcome. The parameter ranges were determined based on preliminary tests to ensure stable and reproducible EDM conditions. These preliminary studies were conducted to identify stable ranges that avoid short-circuit discharges and surface damage. Real-time measurements of voltage and current in the sparking gap were used to verify that the selected values accurately reflected the actual machining conditions:

- Pulse duration *t*_on_: 8–55 µs;
- Pulse interval *t*_off_: 6–75 µs;
- Discharge current *I*_c_: 1.7–5 A;
- Discharge voltage *U*_c_: 25 V;
- Open voltage *U*_0_: 225 V;
- Machining depth: 0.5 mm;
- Tool polarity: negative, with the electrode acting as the cathode and the workpiece as the anode.

A Hartley experimental design was employed, with three independent variables varied across five levels. Table 3 summarises the values of the coded variables used in the experimental design, along with their corresponding actual (uncoded) values. The parameter values correspond to the actual machining conditions measured in the sparking gap, rather than the preset values displayed on the EDM machine. These values correspond to the individual discharge pulses measured in the sparking gap during machining, not cumulative values, and represent stable and reproducible machining conditions.

### 2.6. Methods

After EDM, the samples were sectioned perpendicular to the machined surface using a wire EDM machine Robofil 440 SLP (Charmilles, Geneva, Switzerland) and then hot-mounted in resin. They were subsequently subjected to sequential coarse and fine grinding, followed by polishing to obtain a mirror-like surface. To reveal the microstructure, the samples were chemically etched using Adler’s reagent.

Metallographic observations were performed using an Olympus BX51M optical microscope (Olympus Corporation, Tokyo, Japan) at magnifications of ×200, ×500, and ×1000. High-resolution images were acquired with an Olympus SC50 digital camera (5-megapixel sensor) and analysed using Olympus Stream Essentials software (version 2.4, Olympus Corporation, Tokyo, Japan). For each experimental condition, 15 measurements of the recast layer thickness (WLT) were taken to determine the average value. Microstructural defects, including microcracks and non-uniform resolidified regions, were also evaluated.

This methodology enabled the precise characterisation of the recast layer and microstructural features, ensuring a reliable assessment of the effects of EDM parameters and electrode properties on surface integrity.

## 3. Results and Discussion

### 3.1. Thickness of Recast Layers

The microstructure of the surface layer was examined on samples subjected to EDM with positive polarity using AF-5 and S-180 electrodes, according to the parameters defined in the experimental plan. Table 4 summarises the average recast layer thicknesses measured on the samples machined with the AF-5 and S-180 graphite electrodes.

The measurement uncertainty of the recast layer thickness (WLT) was determined based on 15 repeated measurements for each sample using an Olympus SC50 digital camera (5-megapixel sensor) with ×50 objective magnification. The combined effect of image resolution, optical magnification, and sample preparation was estimated, resulting in a measurement uncertainty of ±0.2 µm for all conditions.

**Figure 1:**
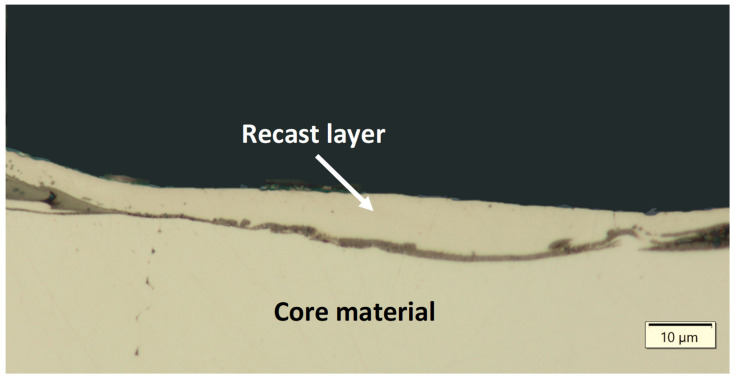
Observation of the recast layer following EDM processing.

Thermal, mechanical, and chemical processes occurring in the surface layer during electrical discharges introduce significant changes to the metallographic structure. Based on the analysis of metallographic section photographs of the tested samples, a recast layer was observed (Figure 1). The recast layer develops from material that melts during the machining process and subsequently resolidifies on the workpiece surface, rather than being completely removed by the dielectric. This layer can significantly influence the surface integrity, including microhardness, residual stress, and corrosion resistance. Based on the analysis of the measured results for the average thickness of the recast layer, significant differences in thickness and continuity of this layer are noticeable, depending on the electrical parameters of the EDM process used, as well as the type of graphite electrode.

The recast layer is characterised by inhomogeneity and discontinuity, and varies in thickness. The discontinuity of the recast layer results from the random occurrence of electrical discharges on the machined surface and the overlapping of individual craters. The thickness of the recast layer is closely dependent on the electrical parameters and the electrical conductivity of the graphite electrodes. For the AF-5 electrode, the average recast layer thickness ranges from WLT = 3.6 to 11.6 μm, while for the S-180 electrode, this range is WLT = 4.6 to 12.8 μm.

For low-energy electric discharges, the average recast layer thickness is lower than for high-energy discharges. Increasing the current intensity (*I*_c_) and discharge time (*t*_on_) increases the amount of eroded material and the depth of the formed craters. The resulting layer is characterised by high thickness variability and discontinuity. The thickness of the recast layer after machining with the S-180 electrode, compared to the AF-5 electrode at the same machining parameters, is on average 2–20% greater in each case. The S-180 electrode has lower resistance and generates more intense electrical discharges in the machining zone than the AF-5 graphite. Consequently, the graphite’s higher electrical conductivity delivers more thermal energy to the workpiece, increasing the thickness of the recast layer.

**Figure 2:**
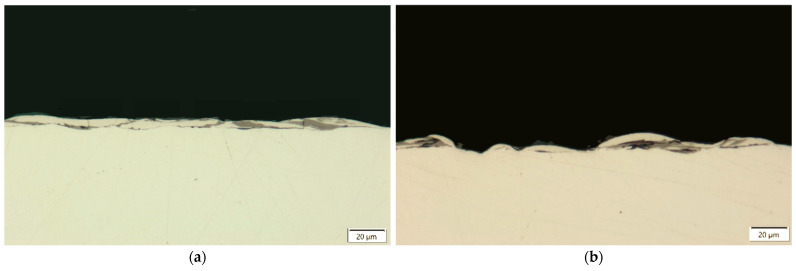
Recast layer formed after EDM under the following conditions: *I*_c_ = 1.7 A, *t*_on_ = 30 µs, *t*_off_ = 37 µs, using (**a**) AF-5; (**b**) S-180 electrodes.

Graphite’s high sublimation temperature, combined with its low apparent density, makes it prone to particle detachment from the electrode during the electroerosion process. This effect is especially pronounced in graphite materials with larger grain sizes and higher porosity. Detached graphite particles are transported across the interelectrode gap by the electric field, where they can interact with the molten material within the plasma channel, contributing to its removal from the resulting spherical crater. This phenomenon has been observed by Torres [55]. Notably, the frequent detachment of larger S-180 graphite grains can impart greater kinetic energy onto the molten pool on the machined surface compared to the finer AF-5 graphite grains. As a consequence of these phenomena, the recast layer generated with the S-180 electrode displays more pronounced variations in thickness and higher discontinuities, highlighting the influence of electrode grain size and detachment behaviour on the surface integrity of the machined material (Figure 2).

**Figure 3:**
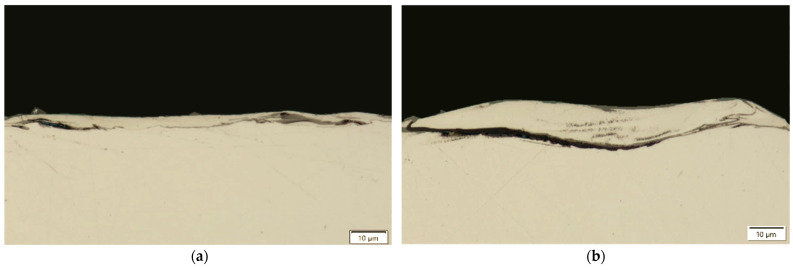
Recast layer after machining with the S-180 electrode under the following parameters: *t*_on_ = 30 µs, *t*_off_ = 37 µs, (**a**) *I*_c_ = 1.7 A; (**b**) *I*_c_ = 5 A.

**Figure 4:**
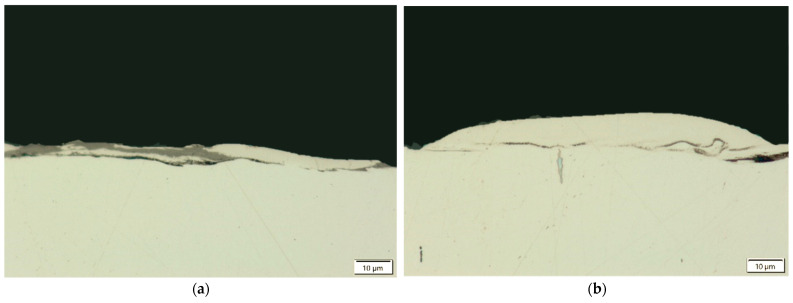
Recast layer after machining with the AF-5 electrode under the following parameters: *t*_on_ = 30 µs, *t*_off_ = 37 µs, (**a**) *I*_c_ = 1.7 A; (**b**) *I*_c_ = 5 A.

Increasing the current intensity from *I*_c_ = 1.7 A to 5 A, while maintaining constant discharge time and time interval between pulses (*t*_on_ = 30 μs, *t*_off_ = 37 μs), results in a noticeable thickening of the recast layer (Figure 3 and Figure 4). This behaviour can be attributed to the higher energy delivered per individual discharge, which increases the amount of material melted and removed. At higher current levels, the recast layer tends to display more pronounced unevenness and discontinuities, reflecting the intensified material transfer and solidification dynamics.

**Figure 5:**
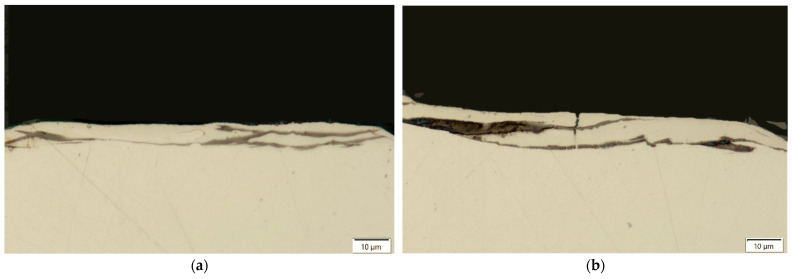
Recast layer after machining with the S-180 electrode under the following parameters: *I*_c_ = 1.7 A, *t*_off_ = 37 µs; (**a**) *t*_on_ = 8 µs; (**b**) *t*_on_ = 55 µs.

**Figure 6:**
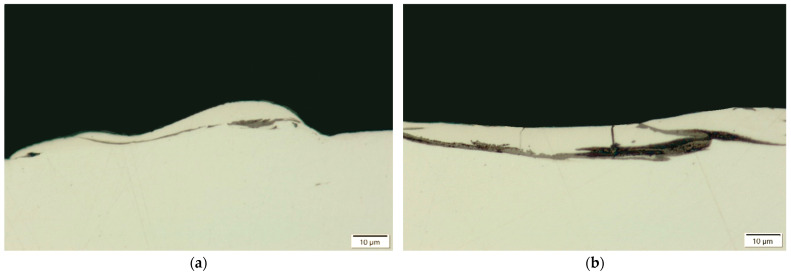
Recast layer after machining with the AF-5 electrode under the following parameters: *I*_c_ = 1.7 A, *t*_off_ = 37 µs; (**a**) *t*_on_ = 8 µs; (**b**) *t*_on_ = 55 µs.

At constant current intensity and time interval (*I*_c_ = 3.8 A, *t*_off_ = 37 μs), increasing the discharge duration from *t*_on_ = 8 μs to 55 μs leads to a substantial increase in the average thickness of the recast layer (Figure 5 and Figure 6). The observed doubling of the layer thickness is attributed to the prolonged interaction of the plasma channel with the workpiece, which generates a more intense heat flux and promotes greater material melting.

The time interval (*t*_off_) was found to have a negligible effect on the average thickness of the recast layer. This parameter does not influence the energy of the electric discharge or the amount of heat transferred to the workpiece. Extending the time interval from *t*_off_ = 6 μs to 75 μs, while keeping the current intensity and discharge time constant (*I*_c_ = 3.8 A, t_on_ = 30 μs), did not produce any significant change in the average layer thickness for either of the tested graphite electrodes.

### 3.2. Microstructural Defects

Based on metallographic observations, structural defects in the recast layer in the form of microcracks were detected. These microcracks result from thermal and tensile stresses generated in the surface layer of the workpiece. The thermal stresses arise from the extremely high temperature of the plasma channel during electrical discharge. A molten material pool forms on the machined surface, which is rapidly cooled and recrystallized by the dielectric fluid flowing through the inter-electrode gap. This rapid solidification causes shrinkage that is resisted by the underlying material. Carbon diffusion from the pyrolysis of the dielectric and the graphite electrode accelerates the shrinkage of the recast layer relative to the base material. When subsurface stresses exceed the tensile strength of the core material, microcracks are formed.

**Figure 7:**
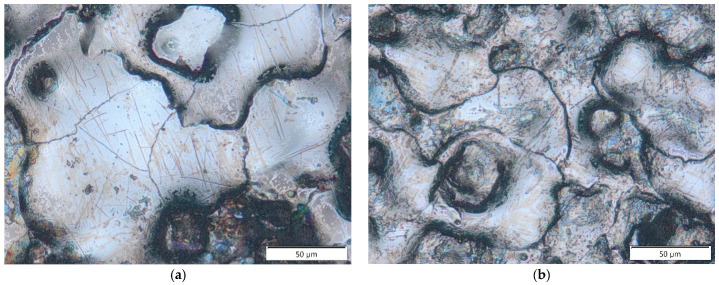
Microcracks on the surface after electrical discharge machining with the AF-5 electrode under the following conditions: (**a**) *I*_c_ = 4 A, *t*_on_ = 41 µs, *t*_off_ = 51 µs; (**b**) *I*_c_ = 2.7 A, *t*_on_ = 17 µs, *t*_off_ = 19 µs.

**Figure 8:**
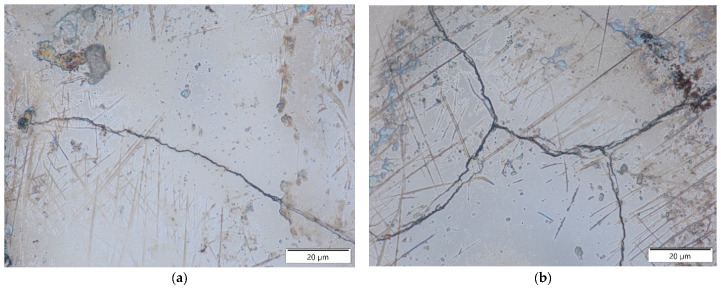
Microcracks on the surface after electrical discharge machining with the AF-5 electrode under the following conditions: (**a**) *I*_c_ = 5 A, *t*_on_ = 31 µs, *t*_off_ = 37 µs; (**b**) *I*_c_ = 3.8 A, *t*_on_ = 55 µs, *t*_off_ = 37 µs.

**Figure 9:**
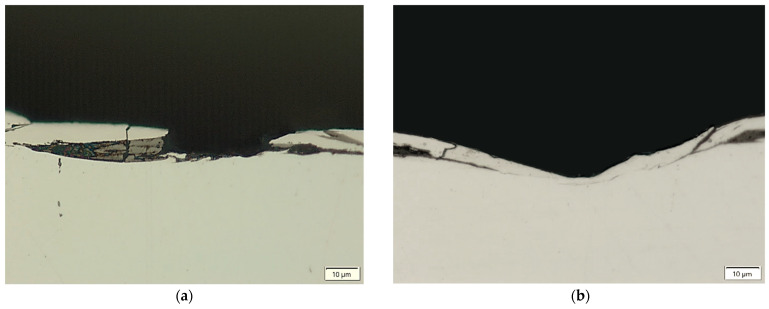
Microcracks in the recast layer after electrical discharge machining with the AF-5 electrode under the following conditions: (**a**) *I*_c_ = 2.7 A, *t*_on_ = 41 µs, *t*_off_ = 19 µs; (**b**) *I*_c_ = 4 A, *t*_on_ = 41 µs, *t*_off_ = 19 µs.

**Figure 10:**
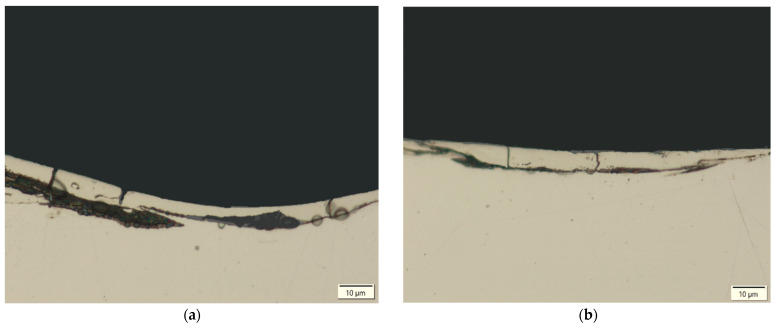
Microcracks in the recast layer after electrical discharge machining with the S-180 electrode under the following conditions: (**a**) *I*_c_ = 4 A, *t*_on_ = 41 µs, *t*_off_ = 51 µs; (**b**) *I*_c_ = *I*_c_ = 4 A, *t*_on_ = 41 µs, *t*_off_ = 51 µs.

Microcracks in the surface layer lead to deterioration of the mechanical properties of the workpiece, including reduced corrosion resistance and fatigue strength, particularly under tensile and dynamic loading conditions. Figure 7, Figure 8, Figure 9 and Figure 10 present examples of microcracks observed in the recast layer. The crack density depends on the machining conditions and the electrode material. Most microcracks are oriented perpendicular to the machined surface, and their depth typically extends to the boundary of the recast layer. In some cases, microcracks propagate parallel to the surface, often leading to the partial detachment of the thin recast layer from the substrate material.

After EDM with the S-180 graphite electrode, a slightly higher density of microcracks was observed in the recast layer compared to machining with the AF-5 electrode. This effect is primarily attributed to the lower electrical resistivity of coarse-grained graphite, which generates higher thermal stresses in the surface layer during EDM.

A strong correlation was found between discharge energy, the thickness of the recast layer, and the number of microcracks. Increasing the discharge current and pulse duration—and thus the energy of a single discharge—resulted in a thicker recast layer with a lower density of microcracks. This may be explained by the concentration of tensile stresses within the thicker recast layer, which promotes the formation of cracks. Conversely, reducing the discharge time and current intensity produced a thinner recast layer that was more susceptible to cracking.

### 3.3. Statistical Models of WLT

The experimental investigation was conducted using Hartley’s experimental design, which incorporated five levels and three input electrical parameters: discharge current (*I*_c_), discharge time (*t*_on_), and time interval between pulses (*t*_off_). To verify the repeatability and reliability of the results, two replicates were performed at the central point of the design matrix. In accordance with the adopted experimental plan, a total of 16 trials were conducted using different combinations of electrical parameters. All experiments were performed under identical machining conditions for both graphite electrodes (AF-5 and S-180).

Based on microstructural observations and measurements of the average recast layer thickness, statistical models of second-degree regression polynomials were developed to describe the influence of the analyzed EDM process parameters on the formation of the layer. Modeling the recast layer thickness enables a quantitative assessment of the relationship between metallographic structural features and the functional properties of components subjected to EDM.

The regression equations were developed in STATISTICA 13.3 using the stepwise regression approach. For each model, the correlation coefficient (*R*), coefficient of determination (*R*^2^), and adjusted coefficient of determination (*R*^2^_adj_) were calculated. The correlation coefficient *R* reflects the strength of the relationship between the input and output variables in the regression analysis—values closer to unity indicate a stronger correlation and a more accurate description of the data variability.

The statistical significance of the correlation was evaluated using the Fisher–Snedecor (*F*) test, where the calculated F-value was compared with the critical value (*F*_kr_). The correlation was considered statistically significant when the ratio *F*/*F*_kr_ ≥ 1 at a significance level of *p* = 0.05. Additionally, the significance of individual regression coefficients was verified using Student’s *t*-test, by comparing the computed *t*-values with their corresponding critical values (*t*_kr_). Terms satisfying the condition *t* ≥ *t*_kr_ were regarded as statistically significant.

Response Surface Methodology (RSM) was employed to develop regression models that describe the EDM process. The developed regression equations for the average recast layer thickness (WLT) exhibited a high correlation coefficient (*R*). At the same time, the *F*/*F*_kr_ ratio significantly exceeded unity in all cases, confirming the reliability of the models. Furthermore, the standard error of estimate remained within acceptably low limits for each equation. Table 5 summarises the regression statistics obtained for the established models.

**Figure 11:**
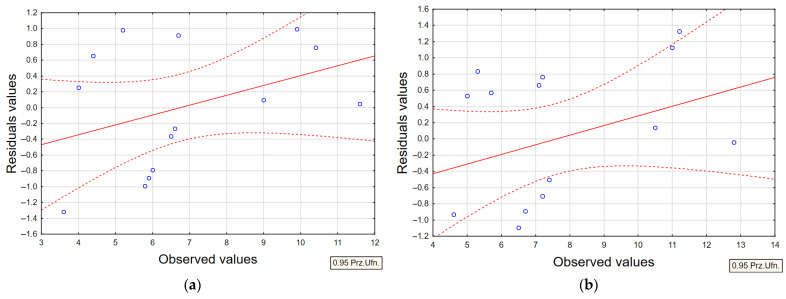
Residuals plotted against observed WLT values for (**a**) AF-5 electrode; (**b**) S-180 electrode.

**Figure 12:**
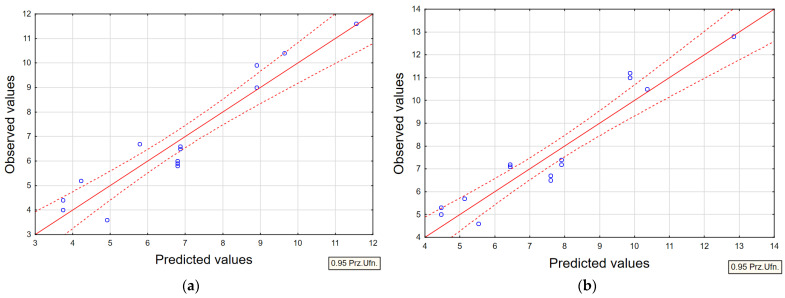
Relationship between observed and predicted WLT values for (**a**) AF-5 electrode; (**b**) S-180 electrode.

For each regression model, residual plots were generated based on observed values. Representative residual distributions for the average recast layer thickness (WLT) using AF-5 and S-180 graphite tool electrodes are shown in Figure 11. Additionally, a scatter plot of observed versus predicted values was created to verify further the accuracy of the developed models (Figure 12). The results indicate high consistency between the experimental and predicted data, with minimal dispersion, confirming the reliability of the models. This analysis allows for the assessment of the goodness of fit between the experimental data and the regression models. Overall, the models demonstrated very low variation between observed and predicted values, highlighting their robustness and the significant influence of the chosen input parameters on the output responses.

After eliminating statistically insignificant terms, the average thickness of the recast layer (WLT) was expressed as a second-order polynomial function of coded variables for discharge current (*X*_1_) and pulse duration (*X*_2_).

The equations representing the thickness of the recast layer obtained after machining with the AF-5 graphite electrode are presented below (1):

WLT_AF-5_ = 6.0 + 0.8 × *X*_1_ + 0.6 × *X*_2_ − 0.3 × *X*_1_ × *X*_2_ − 0.5 × *X*_1_^2^ + 0.2 × *X*_2_^2^ (1)

Similarly, the equations describing the average thickness of the recast layer (WLT) obtained using the S-180 graphite electrode were expressed as second-order polynomial functions (2):

WLT_S-180_ = 7.5 + 1.0 × *X*_1_ + 0.7 × *X*_2_ − 0.4 × *X*_1_ × *X*_2_ − 0.6 × *X*_1_^2^ + 0.3 × *X*_2_^2^ (2)

where

- *X*_1_—coded discharge current;
- *X*_2_—coded pulse duration.

The coded variables were calculated from the experimental values according to the Hartley plan (Table 6).

**Figure 13:**
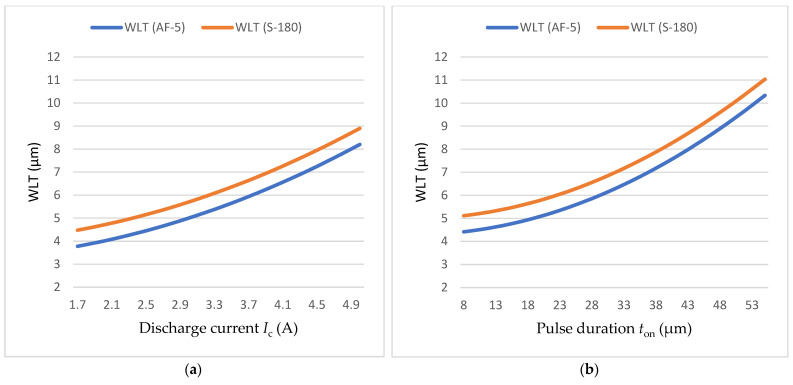
Variation in the average recast layer thickness (WLT) with (**a**) discharge current and (**b**) pulse duration for AF-5 and S-180 graphite electrodes.

The developed predictive models enable the selection of optimal machining conditions based on the desired thickness of the recast layer (WLT) after EDM. The experimental results, when subjected to multicriteria optimisation, can be generalised and applied to modern electrical discharge machines. To further analyse and illustrate the effect of EDM process parameters and graphite electrodes (AF-5 and S-180) on the recast layer thickness (WLT), response surface plots were generated and are presented in Figure 13.

### 3.4. Discussion

The results indicate that the thickness of the recast layer (WLT) in EDM of Hastelloy C-22 is strongly influenced by discharge current (*I*_c_) and pulse duration (*t*_on_), which together determine the total thermal energy delivered to the workpiece. Increasing the current intensity raises the energy in the plasma channel, accelerating melting and evaporation. Prolonged discharge times further increase the volume of molten material, resulting in thicker recast layers. These findings are consistent with previous studies, which have shown that discharge energy is the primary factor controlling recast layer formation [27,35,36,37,45,46,47].

Microstructural observations reveal that microcracks predominantly form perpendicular to the machined surface, with density dependent on both machining parameters and the type of electrode used. Thicker recast layers generally exhibit fewer cracks, likely due to the redistribution of thermal stresses over a larger volume, whereas thinner layers experience more localised stress and higher crack density. Similar trends have been reported in the literature, highlighting the effect of layer thickness and electrode properties on crack formation [48,49]. The S-180 electrode, with lower electrical resistivity and coarser grains, produces slightly thicker recast layers but also a higher density of microcracks compared to the finer AF-5 electrode. This behaviour is attributed to higher thermal energy input and graphite particle detachment, consistent with observations by Torres [55].

Electrode material properties significantly affect recast layer characteristics. Previous studies have shown that tool material influences both layer thickness and microcrack propagation [50], while discharge energy and flushing efficiency are critical for minimising recast layers [45,46]. The developed statistical models confirm that controlling discharge current and pulse duration, along with appropriate electrode selection, allows effective regulation of recast layer thickness and surface integrity. Such control can reduce post-processing operations and enhance the performance of nickel-based superalloys in industrial EDM applications.

## 4. Conclusions

The experimental investigation comprehensively assessed the effects of graphite electrode characteristics (AF-5 and S-180) and key EDM parameters on the average recast layer thickness (WLT) and microstructural integrity of Hastelloy C-22, employing metallographic examinations to observe microcracks and layer uniformity, as well as mathematical models to quantify and predict the relationships between machining conditions and layer formation. The study demonstrated that discharge current, pulse duration, and electrode properties significantly influence WLT and microcrack formation, with higher energy discharges and coarser electrodes producing thicker layers and variable crack densities, providing valuable practical guidance for selecting and optimizing EDM parameters to enhance surface integrity and reduce post-processing, while future research could extend these findings through advanced characterization techniques, real-time monitoring, and exploration of alternative electrode materials to further improve process control and efficiency.

The results led to a series of conclusions based on the experimental data:

1. The discharge current (*I*_c_) and discharge time (*t*_on_) are the dominant factors affecting the average thickness of the recast layer, as they determine the total thermal energy delivered to the workpiece.
2. Higher current intensity (*I*_c_) and longer discharge time (*t*_on_) increase the volume of melted material, resulting in a thicker recast layer due to the resolidification of material not removed from the plasma-formed crater.
3. The density and orientation of microcracks depend on the machining parameters and the type of graphite electrode, with a coarse-grained S-180 electrode producing a slightly higher crack density due to lower electrical resistivity and higher thermal stresses.
4. A strong correlation exists between discharge energy, recast layer thickness, and microcrack formation: higher discharge current (*I*_c_) and longer discharge time (*t*_on_) result in a thicker recast layer with lower microcrack density, whereas lower energy discharges produce thinner layers more prone to cracking.
5. The lower electrical resistance of the S-180 electrode results in more intense discharges, transferring greater thermal energy to the workpiece and producing a thicker recast layer compared to the AF-5 graphite.
6. Detached graphite grains from the electrode, particularly the larger grains of S-180, physically impact the molten material in the plasma channel, which increases local material removal and results in a recast layer with greater thickness variations and discontinuities.
7. The time interval (*t*_off_) primarily affects the stability of electrical discharges and the removal of debris from the interelectrode gap, but it has a negligible impact on the average thickness of the recast layer.
8. The presented results can be further complemented by advanced techniques such as SEM and EBSD to investigate crystallographic orientation, solidification substructure, and fine-scale crack propagation within the recast layer.

## Figures and Tables

**Table 1:** Selected physical properties of POCO graphite grades [53].

Physical Property	Grade AF-5	Grade S-180
Average particle size (µm)	1	10
Shore hardness	87	66
Bulk density (g/cm^3^)	1.80	1.78
Electrical resistivity (µΩ·m)	21.6	13.0
Bending strength (MPa)	117	58

**Table 2:** Nominal chemical composition of Hastelloy C-22 expressed in weight percent (wt.%) [54].

Ni	Cr	Mo	Fe	W	Co	Mn	C
Balance	20.0–22.5	12.5–14.5	2.0–6.0	2.5–3.5	≤2.5	≤0.5	≤0.015

**Table 3:** EDM process parameters and corresponding coded levels.

Level	EDM Parameters		
	Time Interval *t*_off_ (µm)	Discharge Time *t*_on_ (µm)	Discharge Current *I*_c_ (A)
−1.68	6	8	1.7
−1	19	17	2.7
0	37	30	3.8
1	51	41	4
1.68	75	55	5

**Table 4:** Average thickness of the recast layer.

Ex. No.	EDM Parameters			WLT (µm)	
	*t*_on_ (µs)	*t*_off_ (µs)	*I*_c_ (A)	AF-5	S-180
1	17	19	2.7	4.0	5.0
2	17	51	2.7	4.4	5.3
3	41	19	2.7	6.6	7.2
4	41	51	2.7	6.5	7.4
5	17	19	4	6.7	7.1
6	17	51	4	6.7	7.2
7	41	19	4	9.9	11.0
8	41	51	4	9.0	11.2
9	30	37	1.7	5.2	5.7
10	30	37	5.0	10.4	10.5
11	8	37	3.8	3.6	4.6
12	55	37	3.8	11.6	12.8
13	30	6	3.8	5.8	6.5
14	30	75	3.8	5.9	6.7
15	30	37	3.8	5.8	6.7
16	30	37	3.8	6.0	6.7

**Table 5:** Regression summary.

Tool Electrode	Investigated Parameters	Calculated Regression Statistics				
		Ratio *R*	Ratio *R*^2^	Adjusted *R*^2^	Standard Error of Estimation	*F*/*F*_kr_
AF-5	WLT	0.94	0.88	0.86	0.88	2.4
S-180	WLT	0.94	0.88	0.86	0.90	2.5

**Table 6:** Actual and coded levels of EDM process variables according to the Hartley experimental design.

Actual Variable	Symbol	Low Level	Center	High Level	Conversion to Coded Variable
Discharge current (A)	*I*_c_	1.7	3.8	5	$X_{1}=\frac{I_{c}-3.8}{(5-1.7)/2}$
Pulse duration (µs)	*t*_on_	8	30	55	$X_{2}=\frac{t_{on}-30}{(55-8)/2}$

## Data Availability

The original contributions presented in this study are included in the article. Further inquiries can be directed to the corresponding author.
